# A Decade With Sheehan's Syndrome: A Case Report and Personal Experience

**DOI:** 10.1155/crie/6010326

**Published:** 2025-10-12

**Authors:** Kayalvizhi D., Adedeji Yusuf Moradeyo, Bhuvaneswari G.

**Affiliations:** ^1^Department of Child Health Nursing, Saveetha College of Nursing, Saveetha Institute of Medical and Technical Sciences (SIMATS), Chennai, Tamil Nadu, India; ^2^Department of Internal Medicine, Endocrinology Unit, Seychelles Hospital, Victoria, Mahé, Seychelles; ^3^Department of Community Health Nursing, Saveetha College of Nursing, Saveetha Institute of Medical and Technical Sciences (SIMATS), Chennai, Tamil Nadu, India

**Keywords:** adrenal insufficiency, delayed diagnosis, endocrine dysfunction, fertility challenges, hormone replacement therapy (HRT), hypopituitarism, hypothyroidism, metabolic instability, postpartum pituitary necrosis, Sheehan's syndrome

## Abstract

**Objective:**

Sheehan's syndrome (SS) is a rare endocrine disorder caused by ischemic necrosis of the anterior pituitary gland secondary to massive postpartum hemorrhage (PPH) and shock. It commonly leads to multiple hormonal deficiencies, such as adrenal insufficiency, hypothyroidism, and hypopituitarism. Affected individuals may present with signs and symptoms including weight gain or loss, cold intolerance, hair loss, menstrual irregularities, and hypotension. Diagnosis is often delayed due to its nonspecific presentation and overlap with conditions like postpartum depression and chronic fatigue syndrome. This case report highlights the clinical presentation, management challenges, and complications associated with the delayed diagnosis of SS.

**Case Presentation:**

A 35-year-old female was diagnosed with SS in 2016, at the age of 26, following severe PPH and hemorrhagic shock after a vaginal delivery in 2013. Initially, she experienced failure to lactate, followed by amenorrhea, frontal balding, weight loss, hypotension, chronic fatigue, mood swings, and polyuria. For several years, her symptoms were misattributed to postpartum depression, resulting in delayed diagnosis and treatment. A comprehensive endocrine evaluation revealed secondary adrenal insufficiency, central hypothyroidism, and hypogonadotropic hypogonadism, as evidenced by low levels of ACTH, cortisol, TSH, free T4, FSH, LH, and prolactin. Magnetic resonance imaging (MRI) confirmed partial empty sella, supporting the diagnosis of SS. She was started on lifelong hormone replacement therapy (HRT) consisting of hydrocortisone, levothyroxine, and estrogen. She remained highly sensitive to hormone doses, requiring frequent adjustments every other month. Despite modifications, she continued to experience persistent symptoms, including fatigue, hair loss, extreme mood swings, night sweats, and gastrointestinal symptoms such as abdominal discomfort, epigastric pain (worsened on an empty stomach), nausea, vomiting, bloating, and passage of blood in stool, likely related to long-term medication use. Pregnancy was later achieved through ovulation induction using human chorionic gonadotropin (HCG) and human menopausal gonadotropin (HMG). She developed an adrenal crisis during pregnancy, which was managed through hydrocortisone dose modification. The immediate postpartum period was uneventful under treatment, but she required close endocrine follow-up for ongoing metabolic and reproductive concerns.

**Conclusion:**

This case highlights the typical clinical presentation of SS in a postpartum patient, including lactation failure, fatigue, and amenorrhea, which can facilitate early diagnosis of this serious and potentially life-threatening condition. Delayed diagnosis is often linked to prolonged morbidity, affecting both quality of life and fertility. Lifelong HRT, regular endocrinological monitoring, and individualized treatment plans are essential for managing endocrine dysfunction, osteoporosis risk, and cardiovascular complications. Awareness, early screening, and multidisciplinary collaboration among endocrinologists, obstetricians, and reproductive specialists are pivotal in improving long-term outcomes and reducing diagnostic delays.

## 1. Introduction

Sheehan's syndrome (SS) results from ischemic necrosis of the anterior pituitary gland secondary to severe postpartum hemorrhage (PPH). The anterior pituitary is supplied by a low-capacity portal venous system, making it more susceptible to vascular compromise. Following hemorrhage, reduced perfusion of oxygen and nutrients leads to ischemia, cellular necrosis, and subsequent deficiency of anterior pituitary hormones. The typical sequence of hormonal loss, reflecting the differential vulnerability of cell types, is: growth hormone → prolactin → TSH → ACTH → LH/FSH [1].

In contrast, the posterior pituitary gland is rarely affected, as it receives a direct arterial blood supply, making it more resistant to ischemic damage. However, in uncommon cases, diabetes insipidus may occur due to antidiuretic hormone (ADH) deficiency, which may be suggested by the presence of polyuria in this patient.

SS is often underdiagnosed, as its clinical features can mimic postpartum depression, chronic fatigue, or psychological stress [2]. This case underscores the diagnostic challenges of late recognition [3] and highlights the lifelong hormonal deficiencies, fertility issues [4], and the complexity of managing multiple endocrine deficits.

## 2. Case Presentation

A 35-year-old female was diagnosed with Sheehan's Syndrome three years after experiencing severe PPH and hemorrhagic shock following a vaginal delivery in 2013. Initially, she presented with nonlactation and profound fatigue, followed by amenorrhea, hair loss, weight changes, hypotension, mood swings, and frequent urination (Table 1). These symptoms were attributed to postpartum depression for several years, which delayed the correct diagnosis and management. Over time, her symptoms worsened, prompting consultations with multiple specialists, including urologists, gynecologists, and endocrinologists (Table 2).

**Figure 1:**
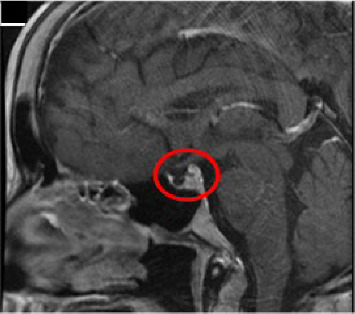
Imaging of the pituitary gland in Sheehan's syndrome: MRI showing empty sella turcica. Magnetic resonance imaging (MRI) is considered the gold standard for diagnosing Sheehan's syndrome. The pituitary gland resides within the sella turcica, a depression in the sphenoid bone. In cases of pituitary necrosis, this space often appears partially or completely empty on imaging. Studies suggest that around 70% of Sheehan's syndrome cases demonstrate a fully empty sella turcica. While MRI provides the most detailed visualization, computed tomography (CT) scans can also aid in the diagnostic process (Figure 1).

In 2016, at the age of 26, she was diagnosed with SS after an extensive endocrine work-up. This included serial measurements of cortisol, thyroid function tests, sodium levels, and vitamin D status, providing insights into her hormonal profile. Magnetic resonance imaging (MRI) of the brain (sella protocol) revealed a partial empty sella, with residual pituitary tissue and loss of the posterior pituitary bright spot, confirming the diagnosis (Figure 1).

She was initiated on lifelong hormone replacement therapy (HRT) for adrenal insufficiency (hydrocortisone 30 mg/day), hypothyroidism (thyroxine 100 mcg/day), and hypogonadism (estrogen [Progynova 10 mg/day] and progesterone [Naturogest 200 mg/day]). Ovulation was induced using human chorionic gonadotropin (HCG) and human menopausal gonadotropin (HMG) injections, resulting in a successful conception.

During pregnancy, she developed an adrenal crisis, presenting with multiple episodes of severe vomiting, which required intravenous hydrocortisone administration—a known complication of adrenal insufficiency during pregnancy in SS. Although the postpartum period remained stable, lifelong endocrine surveillance remains essential.

**Figure 2:**
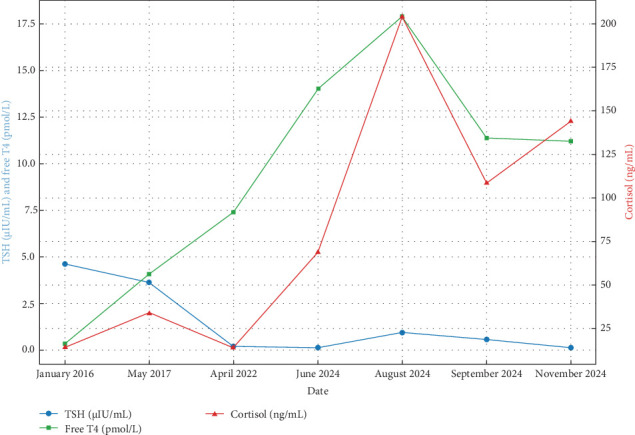
Hormonal trends over time in Sheehan's syndrome. This chart shows fluctuations in TSH, free T4, and cortisol levels between 2016 and 2024. TSH and free T4 are plotted on the left *y*-axis, while cortisol is plotted on the right *y*-axis. The chart illustrates the effect of hormone replacement therapy and dose adjustments over time. *Note:* Data from 2018 to 2021 are unavailable due to loss of personal medical records during a relocation period. Additionally, routine endocrine follow-up was inconsistently documented during this time. Some test intervals also reflect clinical necessity (e.g., symptom worsening, treatment changes) rather than standardized timelines. This emphasizes real-world challenges in maintaining continuous endocrine monitoring in limited-resource settings.

The patient is highly sensitive to fluctuations in hydrocortisone and thyroxine doses as reflected in hormonal variability (Figure 2), often experiencing fatigue, hair loss, low libido, mood disturbances (e.g., irritability, anxiety, and sadness), palpitations, and excessive sweating. She also reports bone pain, osteoporosis, and gastrointestinal side effects such as chronic gastritis, likely due to long-term hydrocortisone therapy (Table 1).

Despite partial improvement, regular hormonal monitoring and medication adjustments have been necessary over a 9-year follow-up period to maintain physiologic hormone levels. She currently undergoes blood tests every 3 months, adjusting doses as needed to limit adverse effects (Table 3). Persistent symptoms such as fatigue, hair thinning, low libido, and intermittent palpitations continue to require attention. These ongoing effects significantly impact her quality of life, underscoring the importance of early diagnosis, timely treatment, and long-term endocrine follow-up.

## 3. Discussion

Postpartum hypopituitarism can result from several conditions, including SS, a rare but serious disorder caused by ischemic necrosis of the pituitary gland [5] following poorly managed severe PPH. The incidence of SS has significantly decreased in developed countries [6] due to improved obstetric care. However, delayed diagnosis remains a major challenge, often due to nonspecific symptoms, late presentation, and misattribution to alternative conditions. Unless patients present early with lactation failure after PPH, symptoms may evolve slowly over months to as long as 10 years, leading to long-term endocrine complications. In many cases, SS is misdiagnosed as postpartum depression, which can significantly delay appropriate management [7]. This delay was evident in the present case, where the diagnosis was made 3 years after delivery.

Although rare in high-resource countries, SS remains a diagnostic challenge in low- and middle-income regions. Studies from India, Africa, and Latin America report diagnostic delays of 5–10 years due to overlapping symptoms with other postpartum disorders, limited endocrine follow-up, and low awareness among healthcare providers [1, 7]. In developed countries, better MRI availability has reduced delays, yet subtle or partial cases may still be overlooked, highlighting the importance of early screening protocols. There are minimal regional data from island nations like Seychelles, making this report an important contribution to the limited literature. The longitudinal follow-up, documentation of treatment responsiveness, and reproductive outcomes in this case provide valuable insight into patient-centered management in underrepresented settings.

SS may present as partial or complete hypopituitarism, with deficiencies in anterior pituitary hormones such as ACTH, thyroid hormones, prolactin, gonadotropins, and growth hormone [8, 9].

The term “SS” refers to pituitary hypofunction arising from ischemic necrosis of the anterior pituitary gland secondary to massive PPH. Risk factors for postpartum hypopituitarism include disseminated intravascular coagulation (DIC), hemophilia, thrombocytopenia, and other bleeding disorders. Clinicians should also be aware of nonobstetric causes such as radiation therapy, empty sella Syndrome, traumatic brain injury, pituitary tumors, neurosurgery, and systemic infections like sepsis. These may mimic SS clinically but differ in etiology and treatment approach.

Characteristic clinical features of SS include failure to lactate, persistent amenorrhea, chronic fatigue, weight changes, and hypotension [10]. Most of these are nonspecific, often leading to misinterpretation as normal postpartum changes or psychiatric conditions [11]. Gradual symptom onset further complicates timely recognition. This case illustrates that even in the presence of severe hemorrhagic shock during delivery, significant hormonal deficiencies may be missed initially, delaying endocrine evaluation (Table 2) [12].

Diagnosis relies on clinical suspicion, laboratory assessment, and imaging studies. Hormonal evaluation should include cortisol, ACTH, TSH, free T4, LH, FSH, and prolactin [13, 14], as deficiencies are common. In this patient, low cortisol and thyroid hormone levels confirmed multiple anterior pituitary hormone deficiencies (Table 3). MRI demonstrated partial empty sella, supporting ischemic pituitary damage [13, 15, 16].

A recent case report detailed a rare and severe presentation of SS diagnosed 6 years after childbirth, in which the patient developed an adrenal crisis, profound hypothyroidism, and massive pericardial effusion as a result of longstanding anterior pituitary insufficiency [17]. This case underscores the potentially life-threatening consequences of delayed recognition and highlights the critical importance of maintaining a high index of suspicion for SS in postpartum women presenting with nonspecific or evolving symptoms. Timely diagnosis and appropriate hormone replacement therapy (HRT) are essential to prevent such serious complications and improve long-term outcomes.

Management of SS requires lifelong HRT. Glucocorticoid replacement with hydrocortisone is essential not only to treat adrenal insufficiency but also to prevent adrenal crisis, especially during stress, illness, or pregnancy. Levothyroxine is used for hypothyroidism [18, 19], and estrogen/progesterone therapy for gonadal failure helps prevent long-term complications such as osteoporosis [20–22] and cardiovascular disease [23]. In this case, frequent dose adjustments were required due to persistent symptoms, illustrating the variability in individual hormone dose responses and the importance of personalized care.

Long-term complications of SS and its treatment include decreased bone mineral density, cardiovascular risks, metabolic disturbances, and psychiatric symptoms [24]. Chronic glucocorticoid therapy increases osteoporosis risk, warranting bone density monitoring and calcium/vitamin D supplementation [21, 22]. Patients frequently report fatigue, mood swings, night sweats, and reduced libido [25–28]. This patient also experienced chronic gastritis, a gastrointestinal side effect of prolonged corticosteroid use [29].

Fertility depends on the extent of pituitary damage. Partial SS may allow near-normal fertility, whereas complete SS significantly impairs reproductive capacity. Amenorrhea and menstrual irregularities are early signs of gonadotropin deficiency. With appropriate HRT, patients can regain reproductive potential and pursue pregnancy. Individualized reproductive counseling is important, taking into account cultural expectations and the emotional impact of infertility.

Women with SS who become pregnant require multidisciplinary monitoring. Pregnancy carries risks, particularly adrenal crisis due to increased hormonal demands. Hydrocortisone dosage should be adjusted during pregnancy and labor to prevent life-threatening complications. With coordinated care involving endocrinologists and obstetricians, favorable maternal and fetal outcomes are possible.

Maternal hormone stability is crucial for fetal growth and neurodevelopment. Poorly controlled maternal hypothyroidism may lead to neurocognitive and developmental delays in the fetus, while prolactin deficiency can result in postpartum lactation failure, depriving the newborn of colostrum, which is vital for neonatal immunity and nutrition.

In some cases, ovulation induction using HCG and HMG injections can restore fertility and lead to successful conception [30]. In this patient, pregnancy was complicated by adrenal crises, necessitating intravenous glucocorticoid therapy, which highlights the importance of adjusting steroid doses during pregnancy to meet increased physiological demands [31].

The integration of emerging technologies, such as wearable hormone monitors and mobile applications, may support real-time hormone tracking and dose optimization, particularly during pregnancy and other vulnerable periods. Existing literature supports a multidisciplinary management approach, involving endocrinologists, obstetricians, and reproductive specialists, to optimize outcomes for both mother and child [32].

Severe PPH, especially when inadequately managed, should raise a high index of suspicion for SS in postpartum women with persistent fatigue, amenorrhea, and lactation failure [32, 33]. This case adds novel patient-specific perspectives, as the patient is also the author, offering a unique narrative medicine dimension. Her case emphasizes diagnostic delays, treatment challenges, psychosocial burdens, and the need for micro-adjustments in hormone dosing. It also highlights the value of longitudinal hormone tracking and the potential of emerging technologies such as wearable and AI-assisted monitoring systems. For instance, the U-RHYTHM system enables continuous cortisol profiling to personalize steroid dosing [34], while wearable nano biosensors now allow noninvasive estradiol monitoring via sweat [35]. Consumer-grade ovulation-tracking wearables are also gaining clinical relevance [36], supporting the integration of mobile apps and AI tools into reproductive care. These advancements, combined with a “Postpartum Endocrine Red Flag Checklist,” may facilitate earlier diagnosis and safer outcomes for high-risk women.

## 4. Conclusion

This report narrates the challenges and consequences of delayed diagnosis in SS, a rare but significant cause of postpartum hypopituitarism [37, 38]. The delay in diagnosis, despite classic features such as fatigue, amenorrhea, and failure to lactate [39, 40] following severe PPH, emphasizes the critical need for heightened clinical vigilance. These symptoms should not be attributed solely to postpartum changes or psychiatric conditions without a comprehensive endocrine evaluation. This case reinforces that the timely diagnosis of SS can significantly reduce long-term morbidity and improve patient outcomes.

Considering the complexity of lifelong HRT [41–43], this case report underscores the potential benefits of technology-based medical support, such as digital hormone monitoring systems or AI-based decision tools for dose optimization.

This case also brings attention to the reproductive challenges in women with SS. The successful conception following ovulation induction and the management of adrenal crises during pregnancy illustrate the importance of coordinated care involving endocrinologists, obstetricians, and fertility specialists [44, 45]. These insights support the integration of innovative healthcare technologies, such as wearable hormone trackers and mobile alert systems, to enhance patient safety during pregnancy.

Importantly, this report demonstrates the value of incorporating patient perspectives in clinical literature. As both subject and author, the patient offers a unique narrative that enriches clinical insight and humanizes medical understanding. This emphasizes the role of narrative medicine in supporting empathetic, patient-centered care.

Ultimately, this case advocates for a multidisciplinary, proactive, and personalized approach to SS. Routine postpartum endocrine screening in high-risk individuals, continuous provider education, and technology-driven care strategies are critical to reduce diagnostic delays and enhance the quality of life. The lessons from this case aim not only to improve clinical care but also to lay the foundation for future research, innovation, and policy development in the management of postpartum endocrine disorders.

## Figures and Tables

**Table 1:** Symptom profile and clinical interpretation in Sheehan's syndrome.

Symptom	Presence in case presentation	Clinical interpretation
Hair loss	Loss of pubic, axillary, eyebrow, body, and scalp hair	Potential indicator of adrenal insufficiency, hypothyroidism, and hypogonadism

Lactation abnormality	Absent following delivery	Prolactin deficiency

Hot flashes	Frequently experienced	May suggest thyroid hormone excess and gonadotrophin deficiency or postsurgical hormonal shifts

Cold intolerance	Noted during colder climates	Potential indicator of secondary hypothyroidism

Mood disturbances	Mood swings, including irritability and low mood	Common in hormonal imbalance—thyroid and cortisol; may mimic psychiatric conditions

Weakness	Generalized weakness reported	Potential indicator of secondary growth hormone deficiency and low cortisol

Extreme fatigue	Persistent fatigue despite treatment	Suggestive of secondary hypothyroidism and possible growth hormone deficiency

Anorexia	Not experienced	Not reported

Nausea	Not experienced	Not reported

Stress	Frequently felt, worsens symptoms	Can act as a trigger for latent Sheehan's syndrome

Mental apathy	Often felt mentally drained or disconnected	Often observed in chronic endocrine disorders

Weight gain/loss	Lost approximately 30 lbs over several months	Indicative of metabolic dysregulation or malabsorption from hormonal imbalance

Gastritis	Diagnosed with chronic gastritis	Common complication from long-term medication use

Libido	Reduced sexual desire	Common in gonadotropin deficiency or hormonal imbalance

Palpitations	Episodes occurred, especially with hormone fluctuation	May be associated with adrenal or thyroid hormone imbalances

Infertility	History of difficulty conceiving, treated with ovulation induction	Common outcome of hypogonadotropic hypogonadism related to pituitary dysfunction

**Table 2:** Timeline of events and diagnostic tests of patient.

Year	Age and key events
2013	Age 23 - Severe postpartum hemorrhage (PPH) and hemorrhagic shock after vaginal delivery, and neonatal loss - Postdelivery complications: persistent fatigue, failure to lactate, amenorrhea, hypotension, and mood swings

2014–2015	Age 24–25 - Symptoms persist: frontal hair loss, weight fluctuations, frequent urination, and worsening fatigue - Misdiagnosed as postpartum depression by multiple specialists

2016	Age 26 - Formal diagnosis of Sheehan's syndrome after endocrinological assessment: - Cortisol: 74.3 µg/dL (low) - TSH: 4.620 mIU/L (abnormal fluctuations) - Free T4: 0.34 ng/dL (low) - FSH, LH, and prolactin: Markedly reduced - MRI (sella protocol): Partial empty sella, thin residual pituitary gland, absence of posterior pituitary bright spot - Initiation of HRT: - Hydrocortisone (30 mg/day) - Thyroxine (100 mcg/day) - Progynova (10 mg/day) - Fertility treatments initiated using HCG and HMG injections - Successful conception - Pregnancy complications: Adrenal crisis requiring IV hydrocortisone -A healthy baby was delivered via timely planned C-section, with the birth weight of 2.3 kg -No complications during C-section -IV hydrocortisone 100 mg/per day was given for 5 days after delivery

2017	Age 27 - Sodium levels: 138 mmol/L (stable but monitored)

2018–2019	Age 28–29 - Postpartum period: Frequent endocrine monitoring and HRT adjustments - Bone pain and osteoporosis risk due to long-term corticosteroid use - Chronic gastritis developed due to extended hydrocortisone therapy

2020–2023	Age 30–34 - Persistent symptoms: Fatigue, libido issues, and hair loss - Frequent dose adjustments required - Sodium levels: 137 mmol/L (2022)

2024	Age 35 - Ongoing 3-monthly endocrine evaluations - Current test results: - Cortisol: 204 µg/dL (fluctuating) - TSH: 0.122 mIU/L (abnormal range) - Free T4: 17.9 pmol/L (adjusted within range) - Sodium: 140 mmol/L (stable but monitored) - Vitamin D deficiency: 9.94 ng/mL

**Table 3:** Hormonal profile with reference ranges and treatment status.

Date	TSH (µIU/mL)	Free T4 (pmol/L)	Cortisol (ng/mL)	Sodium (mmol/L)	Vitamin D (ng/mL)	Treatment given	Dose (thyroxine)	Dose (hydrocortisone)	Treatment status	Clinical improvement
January 2016	4.62	0.34	14.3	139.0	8.0	Thyroxine and hydrocortisone	50 mcg/day	20 mg/day	Baseline	Some improvement
May 2017	3.62	4.07	34.15	138.0	7.2	Thyroxine and hydrocortisone	75 mcg/day	25 mg/day	On-treatment	No significant change
April 2022	0.2	7.4	14.0	137.0	7.4	Thyroxine and hydrocortisone	100 mcg/day	30 mg/day	On-treatment	Significant improvement
June 2024	0.12	14.0	69.0	140.0	7.2	Thyroxine and hydrocortisone	125 mcg/day	35 mg/day	On-treatment	Improved thyroid function
August 2024	0.94	17.9	204	143.0	9.94	Thyroxine and hydrocortisone	100 mcg/day	30 mg/day	On-treatment	Recovery in progress
September 2024	0.56	11.4	109	140.0	8.4	Thyroxine and hydrocortisone	100 mcg/day	25 mg/day	On-treatment	No improvement
November 2024	0.12	11.2	144	141.0	8.1	Thyroxine and hydrocortisone	75 mcg/day	30 mg/day	On-treatment	Stable condition

*Note:* Reference ranges: cortisol: 60–230 ng/mL (morning); free T4: 10–25 pmol/L; sodium: 135–145 mmol/L; TSH: 0.4–4.0 µIU/mL; vitamin D: 20–50 ng/mL. Data from 2018 to 2021 are unavailable due to loss of personal medical records during a relocation period. Additionally, routine endocrine follow-up was inconsistently documented during this time. Some test intervals also reflect clinical necessity (e.g., symptom worsening, treatment changes) rather than standardized timelines. This emphasizes real-world challenges in maintaining continuous endocrine monitoring in limited-resource settings.

## Data Availability

The data supporting the findings of this case report are available from the corresponding author upon reasonable request. To protect patient confidentiality, individual clinical data are not publicly available.

## Supporting Information

Supporting Information File S1: Physician's notes (2016). File S2: Hormone investigation report (2016). File S3: Cortisol report (2016). File S4: MRI report showing partial empty sella (2016). File S5: Endocrinologist's notes with diagnosis and treatment (2016). File S6: Prescription (HMG and HCG to induce ovulation for pregnancy, 2016). FIle S7: TSH report (2017). File S8: Discharge summary (Sheehan's Syndrome delivery, obstetric history). File S9: Endocrinology prescription and dose adjustment (2020). File S10: Biochemistry report on T3, T4, and Cortisol (2021). File S11: Biochemistry report on T3, T4, and TSH (July 2024). File S12: Biochemistry report on Cortisol and TSH (November 2024).
