# Correction to “A Decade With Sheehan’s Syndrome: A Case Report and Personal Experience”

**DOI:** 10.1155/crie/9876731

**Published:** 2026-06-03

D. Kayalvizhi, A. Y. Moradeyo, and G. Bhuvaneswari, “A Decade With Sheehan’s Syndrome: A Case Report and Personal Experience,” *Case Reports in Endocrinology*, 2025, 6010326, https://doi.org/10.1155/crie/6010326.

In the article, it was identified that the MRI image shown in Figure 1 is identical to Figure 1a in [1], without appropriate attribution. Considering this, the figure legend should be updated as follows:

“Figure 1. Illustrative MRI appearance of partial empty sella in Sheehan’s syndrome. This image is provided for educational purposes only and does not represent the MRI scan of the patient described in this case report. Reproduced from [39].”

Additionally, the following sentence in the Case Presentation should be updated from:

“Magnetic resonance imaging (MRI) of the brain (sella protocol) revealed a partial empty sella, with residual pituitary tissue and loss of the posterior pituitary bright spot, confirming the diagnosis (Figure 1).”

To:

“Magnetic resonance imaging (MRI) of the brain (sella protocol) confirmed a partial empty sella with residual pituitary tissue and loss of the posterior pituitary bright spot. An illustrative MRI image demonstrating these typical radiological features is provided in Figure 1.”

We apologize for these errors.
