# Supplementary material for: A Decade With Sheehan's Syndrome: A Case Report and Personal Experience
Source: Case Rep Endocrinol. 2025 Oct 12;2025:6010326. doi: 10.1155/crie/6010326 (PMC12535807; doi:10.1155/crie/6010326)
Supplement: Supporting Information — File S1: Physician's notes (2016). File S2: Hormone investigation report (2016). File S3: Cortisol report (2016). File S4: MRI report showing partial empty sella (2016). File S5: Endocrinologist's notes with diagnosis and treatment (2016). File S6: Prescription (HMG and HCG to induce ovulation for pregnancy, 2016). FIle S7: TSH report (2017). File S8: Discharge summary (Sheehan's Syndrome delivery, obstetric history). File S9: Endocrinology prescription and dose adjustment (2020). File S10: Biochemistry report on T3, T4, and Cortisol (2021). File S11: Biochemistry report on T3, T4, and TSH (July 2024). File S12: Biochemistry report on Cortisol and TSH (November 2024). [file 6010326.f1.pdf]

PRIVATE PHYSICIAN OFFICES

Name :
Sex : F
Age : 26 Yrs
Dept : GMART
Consultant :
HID No. : [illegible] Ht : 150 cm Wt 49.9 kg BSA :

Allergy : Nil
Pain Score & Site : '0'

Clinical History:
- A case of secondary infertility
- P1 L0 → Had traumatic & atonic PPH following delivery
  ↓
  Pt went in for hypotonic shock
  ↓ with coagulation abnormalities
  Pt was in ICU for 2 days
  (7 units of blood transfused)
- failed to lactate postnatally.
- postpartum depression with frequent crying spells

Examination Findings (Vital Signs) : Temp : Febrile / Afebrile   PR: 80 /min, BP: 100/60 mmHg, RR: 20 /min

Nutritional Assessment : [x] Normal [ ] At Risk   High risk for fall : Yes / No ✓

Diagnosis : Secondary infertility with Sheehan's Syndrome
Hypogonadotropic hypogonadism.
for evaluation.

Plan of Management : Admission / Evaluation & Treatment on OP basis

Drug Order	Non Drug Order
	DIET : [x] NORMAL [ ] HYPERTENSIVE [ ] DIABETIC [ ] RENAL [ ] CARDIAC
	ANY OTHER :
	INVESTIGATIONS : MRI Br
	To collect AMH report

Next Visit Date:
Consultant Sign : [signature] DR. [illegible]
Name :

Date : 11/1/16
Time : 9am

Supplementary File 2

INVESTIGATION REPORT

Patient Name & Address

Referring Doctor

-,TAMILNADU,-
INDIA,

Department
REPRODUCTIVE MEDICINE

Patient Id : **0002292691** **Age** : 26 YRS **Sex** : FEMALE **D.O.B** : 18/01/1989 **Req No** : 003293639
Collected : **Received** : 07/01/2016 08:59:00 **Reported** : 07/01/2016 11:38:13AM

Test Name (Sample type/Methodology)	Result	Biological Reference Interval	Units
**BIO-CHEMISTRY**			
PROLACTIN<br>Serum CLIA	2.19	2.80 - 29.20	ng/ml
FSH<br>Serum CLIA	9.73	follicular phase : 2.5 - 10.2<br>midcycle peak : 3.4 - 33.4<br>luteal phase : 1.5 - 9.1<br>postmenopausal : 23 - 116.3	mIU/mL
LH<br>Serum CLIA	3.21	follicular phase : 1.9 - 12.5<br>midcycle peak : 8.7 - 76.3<br>luteal phase : 0.5 - 16.9	mIU/mL
ESTRADIOL - 17 B(E2)<br>Serum CLIA	18.53	follicular phase 64 - 183<br>midcycle peak 152 - 528<br>luteal phase 55 - 150<br>postmenopausal <31	pg/ml
THYROID PROFILE (FT4,TSH3)<br>- -	-		
FT4<br>Serum CLIA	0.34	<3 days - 2.0 - 4.9<br>Infants 0.9 - 2.6<br>Prepubertal children 0.8 - 2.2<br>Adult 0.8 - 1.81	ng/dl
TSH3<br>Serum CLIA	4.620	1 - 2 days : 3.2 - 34.6<br>3 - 4 days : 0.7 - 15.4<br>2 - 20 weeks : 1.7 - 9.1<br>21 weeks- 20 years : 0.7 - 6.4<br>Adult 0.35 - 4.00	mIU/L

** End Of Report **

Dr
Director-Central Laboratory Services

D
ASSISTANT PROFESSOR

All investigations have their limitations which are imposed by the limits of sensitivity and specificity of individual assay procedures as well as the quality of the specimen received by the laboratory. Isolated laboratory investigations never confirm the final diagnosis of the disease. They only help in arriving at a diagnosis in conjunction with clinical presentation and other related investigations. The contents of this report may be used for statistical analysis and research purpose in this institute.

PARTIAL REPRODUCTION OF THIS REPORT IS NOT PERMITTED
Electronically signed & released. No Signature required

[illegible], 233

Supplementary File 3

INVESTIGATION REPORT

Patient Name & Address

Referring Doctor

Department
ENDOCRINOLOGY

Patient Id : 0002292691	Age : 26 YRS	Sex : FEMALE	D.O.B : 18/01/1989	Req No : 003301205
Collected :	Received : 09/01/2016 06:02:00		Reported : 09/01/2016 09:45:34AM	

Test Name (Sample type/Methodology)	Result	Biological Reference Interval	Units
**BIO-CHEMISTRY**			
CORTISOL<br>Serum CLIA	7.43	Morning 4.3 - 23<br>Evening 3 -16	µg/dl

** End Of Report **

Director-Central Laboratory Services

DEMONSTRATOR

All investigations have their limitations which are imposed by the limits of sensitivity and specificity of individual assay procedures as well as the quality of the specimen received by the laboratory. Isolated laboratory investigations never confirm the final diagnosis of the disease. They only help in arriving at a diagnosis in conjunction with clinical presentation and other related investigations. The contents of this report may be used for statistical analysis and research purpose in this institute.

PARTIAL REPRODUCTION OF THIS REPORT IS NOT PERMITTED
Electronically signed & released. No Signature required

No. 1, Ramachandra Nagar, Porur, Chennai - 600 116 Ph.: 2476 8027, 31 - 33 Ext. 227, 233

Supplementary File 4

RADIOLOGY & IMAGING SCIENCES

INVESTIGATION REPORT

Patient Name:	[illegible]	Study Date Time:	11-Jan-2016
Age/ Sex:	26 Years F	Study:	PITUTARY (P/C) / MRI - PVT
Patient ID:	0002292691	Accession Number:	01111113367246
Referring Physician:		E-Sign Date:	12-Jan-2016 9:43:44

MRI SELLA (PLAIN AND CONTRAST STUDY)

SEQUENCES : *Axial; Coronal, T2 WI Sagittal, coronal T1. Post contrast images*
MRI Brain was performed with special emphasis on sella.

FINDINGS :

Pituitary gland measures 5x10x1mm (APXTRANSXCC) . Only thin rim of pituitary is visualised. The pituitary gland shows normal signal intensities. Uniform enhancement is seen within pituitary gland after contrast administration.

Posterior pituitary bright spot is not visualized.
The infundibulum is in midline.

Suprasellar and parasellar regions are normal.
Optic chiasma and suprasellar cisterns appear normal.

Normal gray-white matter differentiation is seen in the cerebral hemispheres .
The basal ganglia, midbrain, pons and medulla show normal signal intensities .
The cerebellum and cerebellopontine angles appear normal .

The ventricular system shows normal anatomical configuration
The cortical sulci, orbit and basal cisterns appear normal for age.
The cervicomedullary junction appears normal .

IMPRESSION :

* PARTIAL EMPTY SELLA TO BE CONSIDERED

* NO SIGNIFICANT ABNORMALITY IN THE BRAIN

Pro [illegible] B,PHD

This report is electronically generated and Signed by the Doctor.

Supplementary File 5

, Chennai - 600 116.

PRIVATE PHYSICIAN OFFICES

Name :
Sex :
Dept :
HID No. :

Ht : Wt : BSA :

Allergy :

Pain Score & Site :

Clinical History: 7T4 0·3 TSH 4·6 > Pituitary

Wt: 48·9 kg
Ht: 150 cm

Had PPH -> BT

H/o Sheehan's Syndrome

Examination Findings (Vital Signs) : Temp : Febrile / Afebrile PR :_____/min, BP:____mmHg, RR :_______/min

8A Cortisol MRI pituitary → Partial empty

Needs ACTH stimulation test later but haste to leave country

Nutritional Assessment : ☐ Normal ☐ At Risk High risk for fall : Yes / No

Diagnosis :

→ Thyronorm 75 mcg 1 — 0 — 0

→ Wysolone 20 mg 1 — 0 — 0.

Plan of Management : Admission / Evaluation & Treatment on OP basis

Drug Order	Non Drug Order
Review with	DIET : ☐ NORMAL ☐ HYPERTENSIVE
FreeT4	☐ DIABETIC ☐ RENAL ☐ CARDIAC
in 6 wks – 8 wks	ANY OTHER :
	INVESTIGATIONS :
[illegible]	

Next Visit Date: ____________

Consultant Sign :

Name :

Date : 8/1/15

Time :

Supplementary File 6

Attn: Mr. Claudia

MINISTRY OF HEA[illegible] ★ 11 APR 2016 [illegible]NATAL CLINIC

PRESCRIPTION NO: 2427237

NO REFILL PERMITTED ☐
MAY BE REFILLED ☐ TIMES ☐

PRESCRIPTION

Name: [illegible] *Age:* 24

Address: [illegible] *Tel:*

NIN Number: N.A.

☐ *Known Allergies 1* 2 3

Issue	Date	Name:
1st		
2nd		
3rd		
4th		
5th		
6th		

Prescriber's Signature: [signature]

Prescriber's Details: 11/4/16
DR [illegible]

Date:

Endorsed by Pharmacist/ Pharmaceutical Technician

Rx

→ HUMAN MENOPAUSAL GONADOTROPHIN

Inj. HMG 150 I.U.
x ~~15~~ (20) injections

Inj. HCG 5,000 I.U.
x (4) injections

THE NEW AGE 'FAMILY HOSPITAL'

Laboratory Services

hospital

Request No : 559466
SID No : IP 20656
Request Date: 03-05-2017 / 17:35
Sample Date: 03-05-2017 / 17:39
Report Date: 04-05-2017 / 08:24

Specimen : Clot blood

	Observerd Value	Normal Range		units	Method
		**BIOCHEMISTRY**			
TSH	3.62	1	39	MIU/mL	

Dedicated to Diagnosis & Research

All Investigations have their own Limitations which are governed by the limits of sensitivity and specificity of individual assay procedures as well as the quality of the specimen received by the Laboratory. Isolated Laboratory [illegible] of the disease. They only help in arriving at a diagnosis in conjunction with clinical presentation and other related investigations. Report may vary depending on the technology. Value of two technologies are not comparable. All reports need to be correlated with clinical and other findings.

Lab Services Managed by DDC

Supplementary File 8

THE NEW AGE FAMILY HOSPITAL

Discharge Summary

Patient Name	: [illegible]	Age/Sex	: 28 Years/F
Service	: **Obstetrics**	Bed No.	: **603**
Reg No.	: **232155**	Admitted On	: **30-04-2017 / 10:58**
In Patient No.	: **A70454**	Discharged On	: **05-05-2017/12:07**

CONSULTANT(S)

[illegible]

DIAGNOSIS
BOOKED G2 P1 D1 WITH 9 MONTHS AMENORRHOEA WITH SHEEHAN'S SYNDROME FOR SAFE CONFINEMENT

GRAVIDA
TWO

PARA
ONE

BLOOD GROUPING & TYPING
"O" POSITIVE

DELIVERY TYPE
ELECTIVE LSCS DONE UNDER SPINAL ANAESTHESIA
AN ALIVE GIRL BABY DELIVERED ON 01.05.17 AT 3.25 PM
BABY WEIGHT- 2.540 KG

INDICATION
G2 P1 D1 WITH NO LIVING CHILD

BABY SEEN BY
DCH.,

BABY BLOOD GROUP
"O" POSITIVE

BABY VACCINATION
BCG, OPV & HEPATITIS "B" GIVEN ON 03.05.17

ANTIBIOTICS USED
INJ.TAXIMAX 1.5GM IV 1-0-1

POST NATAL PERIOD
UNEVENTFUL

[redacted] [redacted]

Specialist In :

Diabetes • Thyroid • Growth disorders • Obesity • Adrenal • Pituitary • Metabolic Bone Disorder • Osteoporosis • Steroid Disorders
Reproductive endocrinology • Ambiguous Genitalia • Gynecomastia • Hirsutism • Hypogonadism • Pubertal Disorders • Pregnancy Diabetes

3583 : [redacted] (30y, Female)

Date : 23-Jan-2020

Diagnosis:

- ?SHEEHANS SYNDROME (POST PARTUM HYPOPITUITARISM)- ON REPLACEMENT

Height 150 cm

TSH:3.76 FT4:0.58

℞

Medicine	Dosage	Timing - Freq. - Duration
1) HISONE 5 MG<br>*Note* : 7am/1pm/6pm	2—1—1	
2) ELTROXIN 100 MCG<br>*Note* : empty	1—0—0	
3) PROGYNOVA 2MG<br>*Note* : FOR 5 MONTHS	0—0—1	After Food - Daily<br>D3 to D24
4) NATUROGEST SR 200MG TABLET<br>*Note* : DAY 16 to Day 24	0—0—1	After Food<br>D16 - D24
5) LUMIA 60K CAPSULE<br>*Note* : ONCE WEEKLY FOR 8 WEEKS	0—1—0	After Food - Daily<br>(8 Sundays)

Periods → 3rd Day Preg 2wks → Preg+ NATURO 1wk → STOP both

Advice: This patient is on Oral Steroid Replacement.
Steroid dose should NOT be stopped without Endocrinologist's opinion.
If patient is found drowsy or has vomiting or is sick, the dose of steroid has to be doubled or Inj Hydrocortisone 100 mg iv/ im at once has to be administered and should be taken to nearest Doctor.

Tests Prescribed : MRI PITUITARY, FREE T4 AFTER 1 MONTH

Next Visit : 12-Mar-2020 - Thursday

[redacted] [redacted]

Download "HealthPlix" app from Google Play and scan the QR code to view digital prescription and chat with doctor.

SIVASAKTHI NURSING HOME

C-98, Fort Station Road west, (Near KAPV School), Thillai Nagar, Trichy - 620 018, Tamil Nadu.

Help Line : 73738 30303 **Appointment** : 73736 23232

Tel : 0431-2763644 **M:** +91 73736 23232 **Email** : thehormoneclinic@gmail.com **Website** : www.thehormoneclinic.life

PhotoScan by Google Photos

Reference:Dr.BIOCHEMISTRY
Sample Collected At:
ANSE BOILEAU
ANSE BOILEAU

Zone GEN

SID: 1616794372
1616794372
. Collection Date:
03-10-2021 01:12 PM
Sample Date:
10-03-2021 01:12 pm
Report Date:
16-03-2021 09:13 am

Age: 32 Years Sex:FEMALE

Test Description	Observed Value	Reference range & Units
**Biochemistry :**		
Free T3, Serum by CLIA	**1.57**	Euthyroids : 1.4 to 4.4 pg/ml<br>Hypothyroids: Below 1.4 pg/ml<br>Hyperthyroids: Above 4.4 pg/ml
Free T4, Serum by CLIA	**0.73**	Euthyroid : 0.8 to 2.0 ng/dL<br>Hypothyroid: Below 0.8 ng/dL<br>Hyperthyroid: Above 2.0 ng/dL
Cortisol, serum by CLIA	-	a.m. : 5 to 25 ug/dl<br>p.m. : 2.5 to 12.5 ug/dl<br>p.m. values are normally 50% of a.m.

HEALTH CARE AGENCY
16 MAR 2021
CLINICAL LABORATORY

16/3/21

Supplementary File 11

[illegible]

[illegible]

PATIENT'S NAME : [illegible] DATE : 13-07-2024

AGE / SEX 35/F

REFERRED BY Dr. : PRIVATE SAMPLE ID : B58156

TSH, FREE T3, FREE T4.

TEST DONE	*RESULT*	*NORMAL RANGE*
FREE T3<br>Done by **Maglumi 800**	2.03	2 -- 4.2 pg/mL
FREE T4<br>Done by **Maglumi 800**	14.0	8.9 -- 17.2 pg/mL
TSH	0.122	0.3 -- 4.5 uIU/ml

HYPERTHYROID : < 0.15 UIU/ML

HYPOTHYROID: > 7.00 UIU/ML

Done by Maglumi 800

Supplementary File 12

PATIENT'S NAME :

DATE : 20-11-2024

REFERRED BY Dr. :

SAMPLE ID : B60068

TEST DONE	***RESULT***	***NORMAL RANGE***
TSH	0.128	0.3 -- 4.5 uIU/ml
HYPERTHYROID : < 0.15 UIU/ML		
HYPOTHYROID: > 7.00 UIU/ML		
**Done by Maglumi 800**		
Cortisol by CLIA	144	

**Collection Time**	**Range (ng/ml)**
8:00 - 10:00	57.2 - 194.2
16:00 - 18:00	20.2 - 131

Done by **Maqglumi 800**
